# Supplementary material for: Comparative Decoding of Physicochemical and Flavor Profiles of Coffee Prepared by High-Pressure Carbon Dioxide, Ice Drip, and Traditional Cold Brew
Source: Foods. 2025 Aug 16;14(16):2840. doi: 10.3390/foods14162840 (PMC12385513; doi:10.3390/foods14162840)
Supplement: Supplementary file 1 [file foods-14-02840-s001.zip › foods-3782917-supplementary.pdf]

Table S1. Aroma sensory scores of the three cold brew coffee

Aroma	TCB	HPCD	ID
Nutty	4.90±1.88[a]	5.37±1.56[a]	4.43±1.98[a]
Smoky	4.43±1.79[a]	4.80±2.02[a]	4.43±2.06[a]
Caramel	4.03±2.09[a]	4.67±1.99[a]	3.93±1.78[a]
Floral	2.13±1.46[a]	1.93±1.05[a]	2.43±1.61[a]
Roasted Potato	2.67±1.60[a]	2.60±1.79[a]	2.23±1.33[a]
Baked	4.07±1.57[a]	4.53±2.18[a]	3.80±1.56[a]
Fruity	2.30±1.82[a]	2.03±1.56[a]	2.37±1.73[a]
Cocoa	4.33±1.99[a]	4.53±1.85[a]	3.93±1.62[a]

The data presented represent mean value ± standard deviation (n ⩾ 3). Significant differences at the $p < 0.05$ level are denoted by distinct lowercase letters within the same row (a is the largest).

Table S2. OPLS-DA models fitting performance values

Indicator	HPCD & ID	HPCD & TCB	TCB & ID
Independent variable fit index (R^2X)	0.937	0.94	0.83
Dependent variable fit index (R^2Y)	0.988	0.964	0.995
Model prediction index (Q^2)	0.966	0.906	0.969
200 Permutation test<br>Q^2 regression line Y-axis intercept	-0.864	-0.859	-1.32

R^2 and Q^2 values > 0.5 usually indicates acceptable model reliability. After 200 permutation tests, the Q^2 regression line Y-axis intercept < 0 suggestes that there is no overfitting of the model, and the model is valid.
